# Acute Effects of Guarana (*Paullinia cupana*) Supplementation on Cognitive Performance, Technical Skills, and Physiological Responses in Amateur Soccer Players

**DOI:** 10.3390/sports14080329

**Published:** 2026-08-03

**Authors:** Safwen Rekik, Mohamed Saifedine Fessi, Sami Hidouri, John Elvis Hagan Jnr, Viorel Petru Ardelean, Vlad Adrian Geantă, Khaled Trabelsi, Wassim Moalla

**Affiliations:** 1 Research Laboratory Education, Motricité, Sport et Santé, EM2S, LR19JS01, High Institute of Sport and Physical Education of Sfax, University of Sfax, Sfax 3000, Tunisia; safwenrekik92@gmail.com (S.R.); saifelfessi@gmail.com (M.S.F.); sami.hidouri@outlook.fr (S.H.); trabelsikhaled@gmail.com (K.T.); wassim.moalla@isseps.usf.tn (W.M.); 2 UPR 4278 LAPEC—Laboratoire de Physiologie Expérimentale Cardiovasculaire, Avignon Université, 84000 Avignon, France; 3 Department of Health, Physical Education and Recreation, University of Cape Coast, Cape Coast F0494, Ghana; 4 Neurocognition and Action-Biomechanics Research Group, Faculty of Psychology and Sports Science, Bielefeld University, D-33501 Bielefeld, Germany; 5 Department of Physical Education and Sport, Faculty of Physical Education and Sport, Aurel Vlaicu University of Arad, 310130 Arad, Romania; vlad.geanta@uav.ro; 6 Department of Movement Sciences and Sports Training, School of Sport Science, The University of Jordan, Amman 11942, Jordan

**Keywords:** caffeinated substances, cognitive function, small-sided games, perceived exertion, mood state, football

## Abstract

This study investigated the acute effects of guarana supplementation on cognitive, technical, physiological, and psychological responses in soccer players prior to, during, and following high-intensity 4 vs. 4 small-sided games (SSGs). Thirty-two male amateur soccer players (20.1 ± 2.9 years) completed a randomized, double-blind, placebo-controlled crossover trial involving guarana (300 mg) and placebo, separated by a 7-day washout. After a 70 min absorption period, participants performed the Trail Making Test (TMT-A/B) and Loughborough Soccer Passing Test (LSPT) before and after a 20 min SSG protocol. Heart rate (HR), HR recovery, perceived physical exertion, perceived mental exertion, mood state, and 24 h wellness were assessed. Guarana significantly improved TMT-A performance compared with placebo (*p* = 0.005, η^2^p = 0.05), whereas no significant differences were observed for TMT-B (*p* = 0.08, η^2^p = 0.02). A significant Group × Time interaction was found for total LSPT performance time (*p* = 0.01, η^2^p = 0.06), driven by a significant technical performance optimization at pre-exercise (*p* < 0.01) that dissipated post-exercise (*p* > 0.05). Guarana significantly increased mean exercise HR (*p* < 0.05) and slowed early recovery kinetics (*p* < 0.05) without altering perceived physical exertion (*p* > 0.05). Concurrently, it significantly reduced perceived mental exertion (*p* < 0.01) and attenuated Total Mood Disturbance (*p* < 0.001). No significant between condition variations were noted for 24 h wellness indices (*p* > 0.05). These findings suggest that acute low-dose guarana administration may improve basic attentional readiness and was associated with improved pre-exercise technical performance, although this performance advantage was attenuated following high-intensity physical exertion.

## 1. Introduction

Top-class soccer players are required to perform approximately 1200 explosive actions per match, including accelerations, decelerations, and rapid changes of direction [1,2]. Players simultaneously engage in complex perceptual-cognitive processes such as sustained attention and rapid decision-making [3]. Given this complexity, and to properly prepare players for game requirements, training practices must replicate both the physiological demands and contextual constraints of match play. Consequently, the small-sided games (SSGs) are frequently used in soccer training to reproduce the physical, technical, and tactical requirements of real competition [4].

Among various SSG formats, 4 vs. 4 is considered to be one of the most effective SSGs that elicit high internal loads, consistently challenging players’ perceived exertion and technical accuracy while maintaining a representative tactical structure [4,5]. Nevertheless, these demands are highly dependent on the pitch dimensions, as variations in the relative pitch area per player significantly manipulate the balance between tactical density and physiological load [4]. Likewise, including stop-ball rules instead of goalkeepers might increase physiological intensity with a higher heart rate (HR) and blood lactate concentration compared to mini-goal formats [6,7]. However, given the balance between physiological demand and game-specific density, the 4 vs. 4 SSG could be observed as an effective format for holistic player development for examining the impact of fatigue on integrated cognitive, tactical behaviours and technical skills in a team-based context [8].

In recent years, nutritional strategies have been increasingly investigated as potential ergogenic aids to enhance both physical and cognitive aspects of soccer performance [9]. Indeed, mental fatigue has been shown to impair soccer-specific decision-making skill [10], highlighting the potential need for targeted nutritional or ergogenic interventions to mitigate these effects. Among these strategies, caffeine supplementation has been widely investigated due to its ergogenic effects on both physical and cognitive performance [10]. The UEFA consensus statement on nutritional supplementation in soccer indicates that caffeine ingestion at doses of 3 to 6 mg/kg of body mass, consumed ~60 min prior to exercise, can be effective in reducing the perception of fatigue, enhancing endurance capacity, improving repeated-sprint performance, and supporting skill execution, fine motor control, and cognitive function [11]. Recent evidence has demonstrated that caffeine ingestion can significantly enhance repetition velocity and explosive power output during high-intensity tasks [12], highlighting its potential to maintain explosive sports performance. Furthermore, psychomotor efficiency and basic cognitive abilities have been shown to clearly differentiate professional athletes from non-athletes, underscoring the critical dependency of sport-specific skill execution on underlying executive functions [13]. Mechanistically, these ergogenic attributes of caffeinated compounds are primarily mediated through their actions as non-selective adenosine receptor antagonists, which effectively elevates central nervous system drive and blunts the subjective perception of effort during exercise [14,15].

Guarana (*Paullinia cupana*) is a plant-based source of caffeine and a native fruit of the Amazon basin which has been used as a traditional medicine by indigenous populations for centuries [16]. Guarana effects are mainly explained by its action on the central nervous system. Specifically, caffeine blocks adenosine A_1_ and A_2A_ receptor, which antagonizes adenosine-mediated inhibition and promotes the release of excitatory neurotransmitters such as dopamine and norepinephrine [14]. However, guarana differs from pure caffeine because its seeds also contain additional bioactive compounds, including methylxanthines (e.g., theobromine, theophylline) and various polyphenolic compounds. These constituents may influence caffeine pharmacokinetics and potentially prolong its stimulant effects. Indeed, the phytochemical profile of guarana, including tannins and polyphenols, may slow caffeine metabolism and extend the ergogenic window beyond that of pure caffeine, potentially producing a more sustained stimulant effect [16]. Nevertheless, recent evidence suggests that the ergogenic effects of guarana may largely be explained by its caffeine content. For example, Talik et al. [17] compared guarana with caffeine supplementation and concluded that the ergogenic effects of guarana may be primarily attributable to its caffeine content, with no additional benefit from its other constituents.

Earlier studies in non-sport populations showed that supplementation with guarana can improve cognitive performance and reduce fatigue [18]. More recently, research has begun to investigate its potential effects in sport-specific contexts. Estrázulas et al. [19] showed that guarana supplementation improved intermittent exercise performance (Yo-Yo IR1) in young soccer players, although no improvements were observed in sprinting or agility performance. Similarly, Gurney et al. [20] reported that guarana supplementation may improve reaction time as well as alertness before and after maximal cycling. However, findings remain inconsistent. For instance, in their systematic review and meta-analysis, Hack et al. [21] revealed small improvements in reaction time following guarana supplementation, with no significant improvement in response accuracy. Furthermore, recent evidence suggests that differences in supplementation protocols, particularly with respect to dosage, may contribute to the variability of outcomes reported across studies [22].

Despite the growing interest in guarana as a natural ergogenic aid, its effects on the integrated demands of soccer performance remain largely unexplored. To date, most studies have examined cognitive outcomes or physical performance in isolation. However, soccer performance emerges from the interaction between physiological strain, cognitive processing, technical execution, and psychological state during high-intensity intermittent exercise. Accordingly, the aim of the current study was to investigate the effects of acute guarana supplementation on cognitive function, technical skill, physiological responses, and psychological state before, during, and after high-intensity 4 vs. 4 SSG in soccer players. We hypothesized that acute guarana supplementation would influence cognitive function and performance in soccer players compared to placebo.

## 2. Materials and Methods

### 2.1. Participants

The required sample size was determined a priori using G*Power software (version 3.1.9.6; Kiel University, Kiel, Germany). Based on a randomized, double-blind crossover design with two conditions (guarana and placebo) and two time points (pre- and post-exercise), a power analysis was conducted using the F-test. A statistical power of 0.90 was selected, rather than the conventional threshold of 0.80, to reduce the risk of Type II error and ensure adequate sensitivity to detect potentially subtle changes in cognitive and technical performance within the crossover design. Assuming a moderate effect size (f = 0.25), the analysis indicated that a minimum of 30 participants was required. A total of 40 male soccer players were initially screened for eligibility. Thirty-two healthy outfield players from local and regional leagues met the eligibility criteria and voluntarily participated in the study (mean ± SD; age: 20.1 ± 2.9 years; body mass: 76.4 ± 7.1 kg; height: 180.2 ± 5.5 cm; body mass index [BMI]: 23.5 ± 1.9 kg/m^2^). Participants had 5.0 ± 1.2 years of competitive soccer experience and trained at least four times weekly, plus one official match.

The selection was based on the following inclusion criteria: (1) age between 18 and 30 years; (2) a minimum of 5 years of competitive soccer experience; (3) a training frequency of 4 sessions per week; (4) status as an outfield player; and (5) reported no known hypersensitivity or previous adverse effects related to caffeine, guarana, or other stimulant-containing products. Participants were excluded if they: (1) reported any diagnosed medical, behavioral, or learning conditions that could influence cognitive or physical performance; (2) had any musculoskeletal injuries within the 3 months prior to the study; or (3) were using other ergogenic aids or medications during the experimental period. Habitual caffeine intake was not quantitatively assessed. However, participants were instructed to refrain from consuming caffeine-containing foods, beverages, supplements, and energy drinks for 24 h before each experimental session to minimize acute caffeine exposure and potential carryover effects between conditions. Although this washout period reduced the likelihood of acute caffeine-related effects, it did not allow control for potential interindividual differences in habitual caffeine consumption or tolerance [11,18]. Prior to the beginning of the protocol, all participants were fully informed of the objectives, experimental procedures, and their right to withdraw at any time. Written informed consent was obtained from all participants. This research was not prospectively registered in a public trial registry. The study was conducted in accordance with the Declaration of Helsinki and approved by the Institutional Committee of Jendouba University (approval code: PH-121-2021).

### 2.2. Study Design

This study utilized a randomized, double-blind, placebo-controlled crossover design. To eliminate inter-individual variability, each participant served as their own control. Participants were randomly assigned to two counterbalanced groups (*n* = 16) to determine the sequence of experimental conditions: Guarana (GUA; 300 mg) or Placebo (PLA). Both experimental visits were separated by a minimum seven-day washout period to ensure total metabolic clearance and prevent carryover effects. Randomization was conducted by an independent researcher not involved in data collection. Allocation was hidden using sequentially numbered, opaque, sealed envelopes, opened only on the morning of the first trial to maintain double-blind integrity.

### 2.3. Procedure

**Figure 1:**
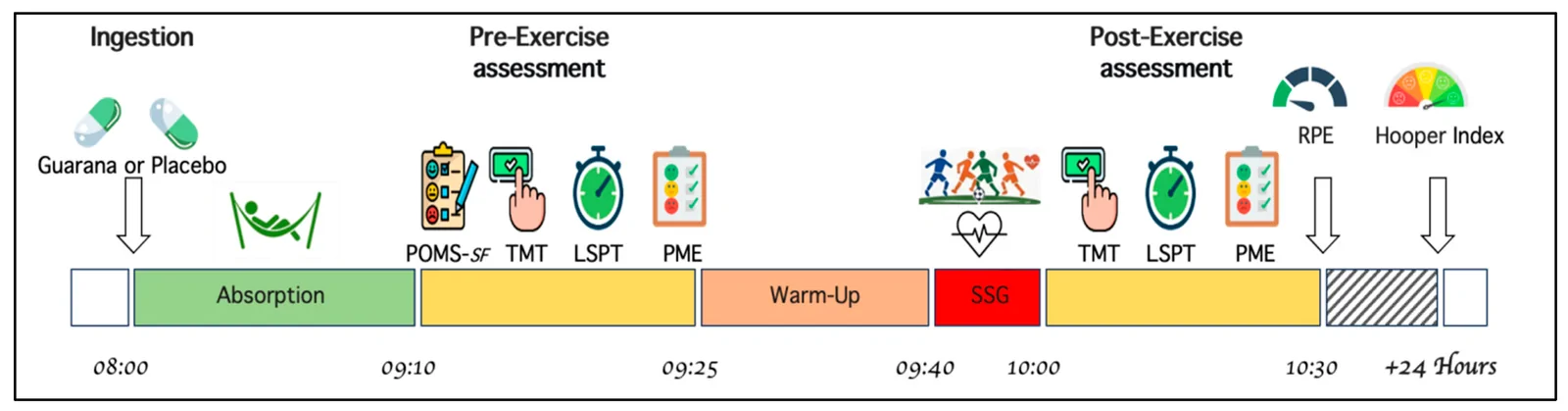
Experimental timeline of a single testing session. TMT = Trail Making Test; LSPT = Loughborough Soccer Passing Test; POMS-SF = Profile of Mood States Short Form; PME = Perceived Mental Exertion; SSG = Small-Sided Game; RPE = Rating of Perceived Exertion.

To ensure full protocol understanding and minimize potential learning effects, participants first completed a dedicated familiarization session where they practiced the complete testing sequence, including the Trail Making Test (TMT-A and B), the Loughborough Soccer Passing Test (LSPT), and the 4 vs. 4 small-sided game (SSG). Three days prior to the first experimental trial, a baseline assessment was conducted at 08:00 AM to establish pre-supplementation reference values for all cognitive, technical, and psychological variables. All subsequent experimental testing sessions were strictly conducted at 08:00 a.m. under standardized in-door microclimatic conditions (mean temperature: 21.4 ± 0.6 °C; relative humidity: 52 ± 4%) to minimize the confounding influence of circadian rhythms, avoid interactions with other nutrients, and maximize supplement absorption efficiency. As detailed in the experimental timeline (Figure 1), upon arrival at the climate-controlled indoor training facility at 08:00 a.m., participants were immediately fitted with HR monitors (Polar H10, Polar Electro Oy, Kempele, Finland) to ensure optimal electrode contact and proper equipment stabilization.

Following the baseline HR assessment, participants ingested either a single 300 mg guarana capsule or an identical-looking placebo. They then completed a standardized 70 min quiet rest period before commencing the cognitive and physical assessments. This interval was selected based on pharmacokinetic evidence indicating that peak plasma caffeine concentrations after guarana ingestion typically occur approximately 60–90 min post-ingestion [16].

At the pre-exercise assessment phase (09:10 a.m.), participants completed the primary battery of tests in a fixed sequence. Then, following a standardized 15 min warm-up supervised by a certified strength and conditioning specialist—consisting of 5 min of low-intensity continuous jogging, 5 min of dynamic stretching (including walking lunges, leg swings, and skipping drills), and 5 min of soccer-specific technical and agility exercises (comprising short progressive accelerations and paired short-distance passing)—participants performed the 4 vs. 4 intermittent small-sided game (SSG) protocol.

The SSG protocol consisted of four 4 min bouts separated by 2 min passive recovery intervals, conducted on a 40 × 30 m artificial turf pitch. HR was monitored continuously throughout the entire protocol, and the Rating of Perceived Exertion (Borg CR10; 0–10) was recorded exactly 30 min post-exercise. Immediately following the final SSG bout (~10:02 a.m.), TMT, LSPT, and Perceived Mental Exertion (PME) were reassessed to evaluate the combined effects of acute fatigue and supplementation. Finally, subjective well-being status was assessed 24 h later using the Hooper Index.

Participants were tested in two cohorts over consecutive days to ensure identical timing. Throughout the study, training loads were strictly maintained at a moderate level, and no official matches were performed. To further ensure data integrity, participants were required to completely abstain from strenuous exercise for 48 h prior to each experimental trial. Participants were instructed to maintain their usual dietary habits but strictly avoid all caffeine-containing products (e.g., coffee, tea, energy drinks, chocolate) for 24 h before each session. On the morning of each trial, participants arrived at the facility in a fasted state (at least 8 h postprandial) to ensure standardized absorption of the capsules, with only ad libitum water intake permitted until the start of the protocol [18]. All trials were supervised by the same investigators, and participants were required to wear the same football boots during both visits.

### 2.4. Guarana and Placebo Supplementation Protocol

Participants received a single oral dose of either 300 mg of standardized guarana extract (Guarana INSIDE; Activ’Inside, Beychac-et-Caillau, France) or an identical placebo capsule. The manufacturer verifies that the extract is standardized to 26% caffeine content by dry weight, which corresponds to approximately 78 mg of pure caffeine per capsule. Given inter-individual differences in body mass, this absolute fixed dose translated into a relative intake ranging from 0.85 to 1.21 mg/kg across participants, thereby maintaining a low-dose ergogenic range across the entire cohort. To preserve the integrity of the double-blind design, both the guarana and placebo capsules (containing microcrystalline cellulose) were identical in appearance and were opaque, odorless, and tasteless. The dosage was selected to achieve an optimal balance between cognitive effectiveness and safety, remaining well below recognized toxicity thresholds and consistent with reported tolerance levels [18,23]. Although this dose is lower than the commonly reported ergogenic range for maximal physical performance (3–6 mg/kg), it is sufficient to antagonize central adenosine receptors, thereby enhancing alertness and cognitive processing speed [14]. Participants were closely monitored for potential adverse effects throughout the experimental sessions, and no side effects were reported following guarana supplementation compared with the placebo.

### 2.5. Trail Making Test (TMT)

Executive functions were assessed using the Trail Making Test (TMT), administered in a quiet, standardized environment on A4-sized sheets to minimize external distractions. The objective of TMT Part A was to measure visuomotor processing speed and visual scanning by requiring participants to connect 25 encircled numbers in ascending order as rapidly as possible. TMT Part B assessed complex cognitive flexibility, set-shifting, and divided attention by requiring the alternating connection of numbers and letters in ascending and alphabetical sequences [3]. Performance for both TMT-A and TMT-B was quantified as the total completion time recorded to the nearest 0.01 s. To maintain protocol integrity, any errors were immediately identified by the investigator, and participants were required to correct the mistake before proceeding. This correction time was inherently included in the final completion score, providing a composite measure of both speed and cognitive control.

### 2.6. The Loughborough Soccer Passing Test (LSPT)

The LSPT was used to assess technical proficiency and decision-making under time pressure within a standardized 12 × 9.5 m grid. This assessment has demonstrated high reliability for soccer-specific skills (ICC = 0.88–0.95) [24]. To simulate match-representative demands, a maximum of two ball-touches per possession was strictly imposed. This constraint increases physiological intensity and technical engagement while maintaining higher passing success rates compared to one-touch play [24,25]. For each assessment point, performance was quantified using Total Performance Time (TPT), calculated as the sum of execution time and penalty seconds (TPT = RT + PT). Penalty Time (PT) was incurred for missing a target (+5 s), hitting a cone (+2 s), exceeding two touches (+2 s), passing outside the area (+3 s), or failing to complete the sequence on time (+5 s). Performance times were recorded using a high-precision digital timing system (Omega, Swiss Timing, Megatek, France; software version v2.1)) to ensure millisecond accuracy (0.001 s).

### 2.7. Small-Sided Game (SSG)

This configuration allowed all athletes (16 per cohort) to engage in high-intensity exercise simultaneously, ensuring identical environmental and circadian conditions [1,25]. The protocol consisted of four 4 min bouts separated by 2 min passive recovery intervals [1]. To ensure a continuous work rate, a “stop-ball” rule was implemented; investigators immediately introduced a new ball whenever the active ball left the playing area [26]. Investigators did not provide tactical instructions or verbal encouragement during the games. HR was continuously recorded at 5 s intervals during SSG using Polar H10 chest straps. Data were transmitted in real time to the Polar Team Pro application. Ratings of Perceived Exertion (RPE) were collected 30 min after completion of the SSG using a modified 10-point Borg scale (CR10) [26]. Assessments were administered individually using digital tablets in a quiet area to minimize social bias.

### 2.8. Perceived Mental Exertion (PME)

Perceived Mental Exertion (PME) was assessed using a numerical rating scale ranging from 0 (“no mental effort at all”) to 10 (“maximal mental effort imaginable”). Participants were instructed to rate the amount of mental effort associated specifically with concentration and attentional demands, while explicitly excluding perceptions of physical effort [27].

### 2.9. Profile of Mood States Short Form (POMS-SF)

The Profile of Mood States was administered to assess acute fluctuations in emotional state using its French short form (POMS-SF), which has demonstrated good reliability and validity [28]. The assessment includes 37 items and six sub-scales. The measure of Total Mood Disturbance (TMD) was obtained by adding the five negative mood scales and subtracting the vigor-activity scale.

### 2.10. Wellness and Recovery Status

Wellness was monitored 24 h after each session using the Hooper Index [29]. Participants responded regarding: (i) quality of sleep, (ii) perceived stress, (iii) fatigue, and (iv) muscle soreness (DOMS). Each item was scored on a 1–7 scale, where lower scores indicate better wellness.

### 2.11. Statistical Analysis

All statistical analyses and data curation were performed using R (version 4.2.1; R Core Team, 2023). Linear Mixed-effects Models (LMMs) were fitted using the lme4 package, with *p*-values obtained via lmerTest. LMMs were prioritized over traditional ANOVA to better account for the crossover design’s inherent dependencies and missing data, treating Group (Guarana vs. Placebo) and Time as fixed factors, and Subject as a random intercept. All statistical inferences for repeated-measures outcomes (TMT, LSPT, HR) were derived from these LMMS, with main effects and interactions evaluated via Type III Wald F-tests using Satterthwaite’s approximation for degrees of freedom. Post hoc pairwise comparisons were conducted using *emmeans* with Bonferroni adjustment to control the family-wise error rate. Specifically, alpha levels were adjusted for each outcome family: POMS (0.008), TMT (0.025), LSPT (0.017), and HR (0.025). For single-measurement outcomes (e.g., Hooper Index, RPE), normality was assessed via Shapiro–Wilk tests. Comparisons were performed using paired *t*-tests or Wilcoxon signed-rank tests (re-porting effect size Z/√N) depending on distributional assumptions. Effect sizes for LMMs were reported as partial eta squared (η^2^p), accompanied by 90% confidence intervals (CIs). Significance for all other tests was set at *p* < 0.05, and all tests were two-tailed.

## 3. Results

### 3.1. Cognitive Performance (TMT-A and TMT-B)

**Figure 2:**
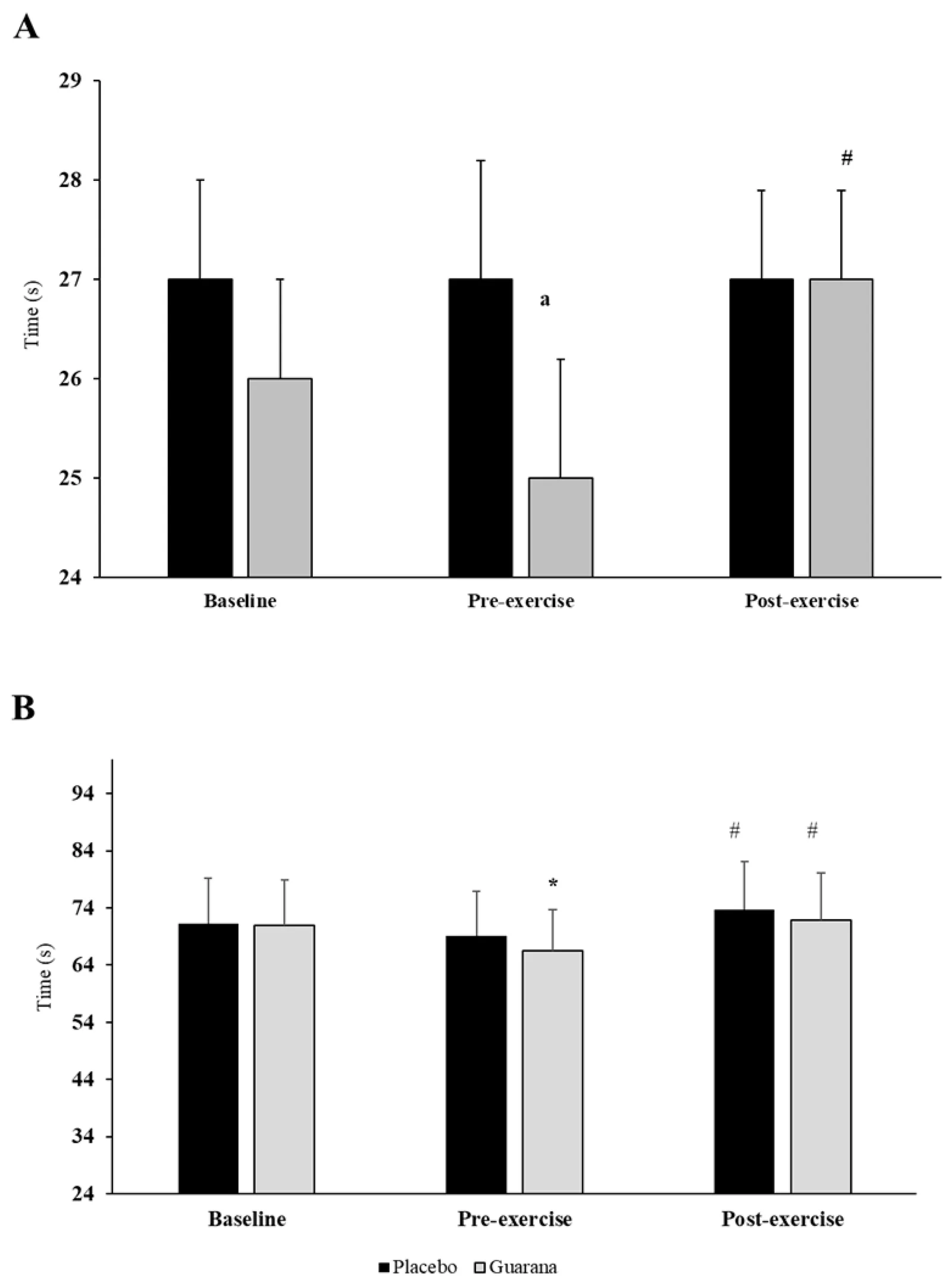
Completion times for the Trail Making Test Part A (**A**) and Part B (**B**) at baseline, pre-exercise, and post-exercise for guarana and placebo conditions. a: significantly different between conditions (*p* < 0.05); *: significantly different from baseline within the same condition (*p* < 0.05); #: significantly different from pre-exercise within the same condition (*p* < 0.05).

LMMs results for Group, Time, and Group × Time interaction effects across cognitive, technical, and physiological outcomes are summarized in Table 1. For TMT-A, the analysis revealed a significant main effect of Group (F = 7.97, *p* = 0.005, η^2^p = 0.05) and Time (F = 4.16, *p* = 0.02, η^2^p = 0.05), with no significant Group × Time interaction (F = 0.44, *p* = 0.64, η^2^p = 0.01; Figure 2A). Guarana administration resulted in an overall improvement of approximately 3.7% faster completion times compared to the placebo, with the largest between-condition difference observed at pre-exercise (7.4% faster under the Guarana condition). For TMT-B, no significant main effect of Group was observed (F = 3.07, *p* = 0.08, η^2^p = 0.02). However, a significant main effect of Time was found (F **=** 10.37, *p* < 0.01, η^2^p = 0.12), while the Group × Time interaction was not statistically significant (F = 0.55, *p* = 0.57, η^2^p = 0.01; Figure 2B). Overall, TMT-B completion time improved by approximately 4.2% from baseline to pre-exercise across both conditions, followed by a deterioration in performance after the small-sided games.

### 3.2. Technical Performance (LSPT)

**Figure 3:**
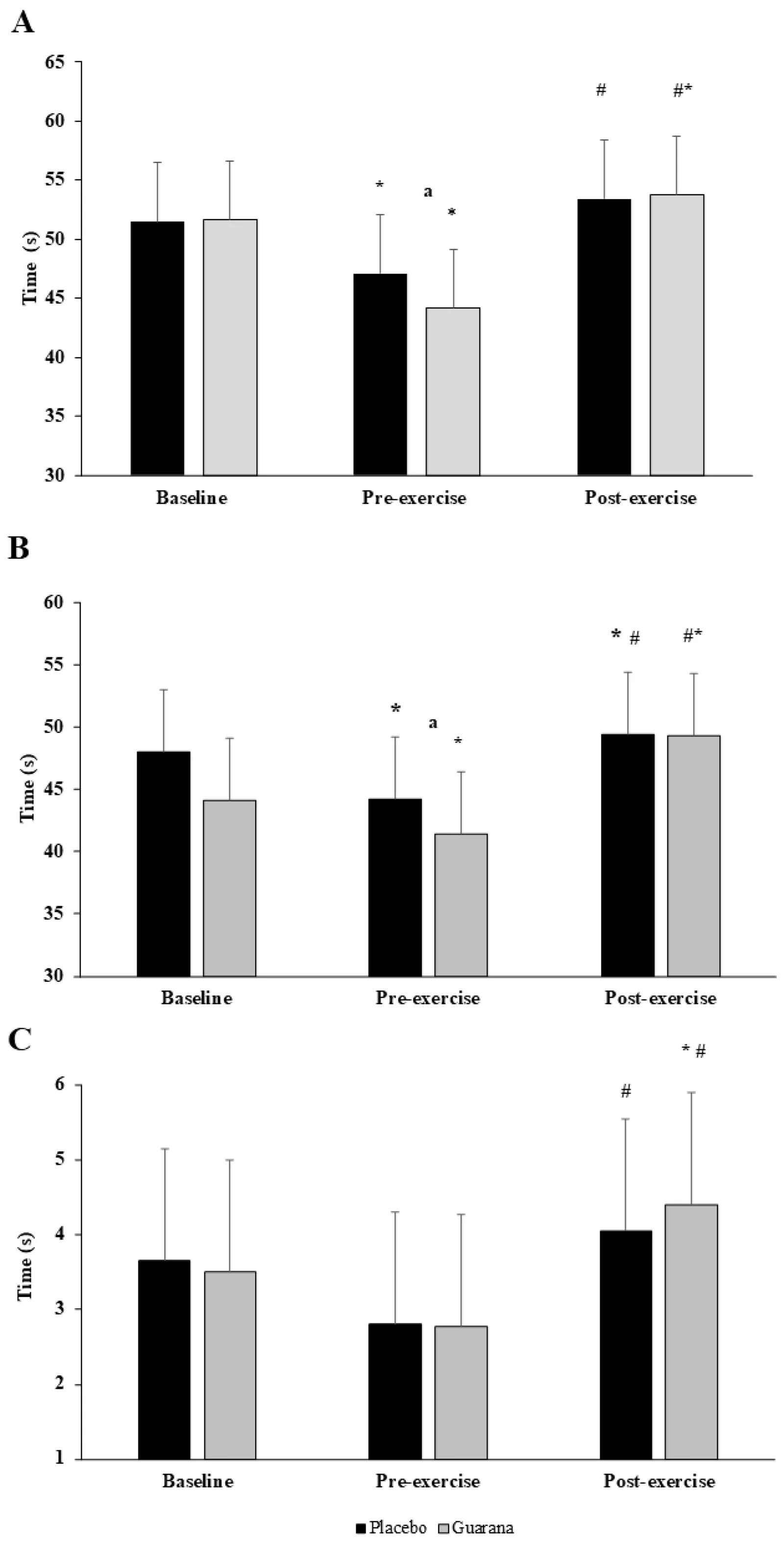
Technical performance during the Loughborough Soccer Passing Test showing (**A**) Total Performance Time, (**B**) Raw Time, and (**C**) Penalty Time at baseline, pre-exercise, and post-exercise for guarana and placebo conditions. a: significantly different between conditions (*p* < 0.05); *: significantly different from baseline within the same condition (*p* < 0.05); #: significantly different from pre-exercise within the same condition (*p* < 0.05).

For LSPT total performance time, the LMM revealed no significant main effect of Group (F = 3.03, *p* = 0.08, η^2^p = 0.02), but demonstrated significant main effects of Time (F = 103.41, *p* < 0.001, η^2^p = 0.57) and a significant Group × Time interaction (F = 4.96, *p* = 0.01, η^2^p = 0.06; Figure 3A). Post hoc pairwise comparisons indicated no baseline differences between conditions. At the pre-exercise assessment, total performance time was significantly shorter in the guarana condition than in the placebo condition (6.2% faster completion time; *p* < 0.01), indicating an acute enhancement in soccer-specific psychomotor performance. However, no between-condition differences were observed after exercise (0.5% difference; 53.75 ± 3.67 s vs. 53.46 ± 6.36 s; *p* > 0.05), suggesting that the pre-exercise technical advantage conferred by guarana was eliminated following the high-intensity demands imposed by the small-sided games. Analysis of the LSPT components revealed that the LMM for Raw Time showed significant main effects of Group (F = 6.63, *p* = 0.01, η^2^p = 0.04) and Time (F = 119.05, *p* < 0.001, η^2^p = 0.61), together with a significant Group × Time interaction (F = 7.00, *p* = 0.01, η^2^p = 0.08; Figure 3B). In contrast, Penalty Time exhibited only a significant main effect of Time (F = 20.43, *p* < 0.001, η^2^p = 0.21), whereas neither the main effect of Group (F = 0.46, *p* = 0.50, η^2^p = 0.00) nor the Group × Time interaction (F = 0.37, *p* = 0.69, η^2^p = 0.00) reached statistical significance (Figure 3C).

### 3.3. SSG Physiological and Perceived Load

**Figure 4:**
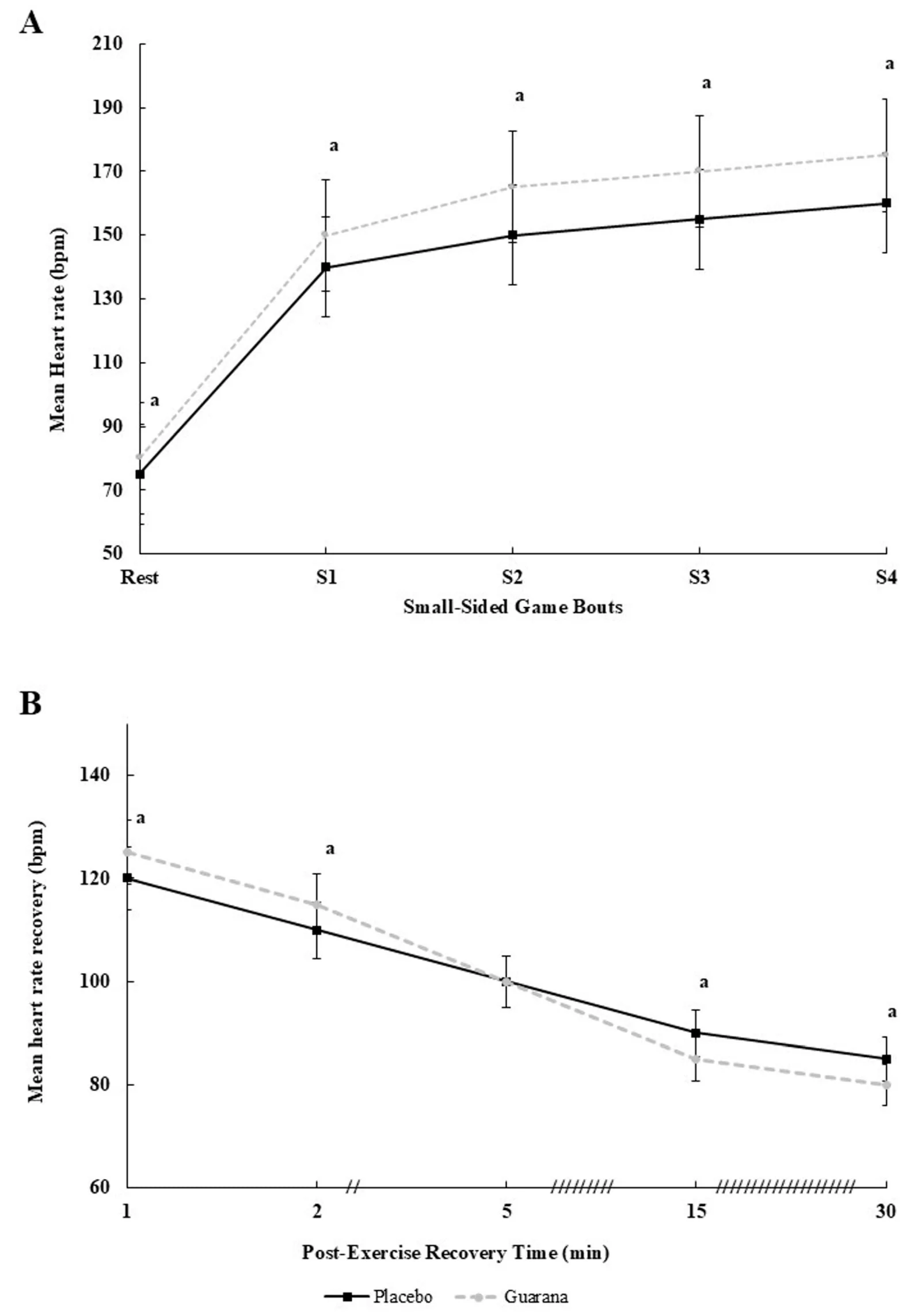
Heart rate kinetics during rest and SSG sessions (**A**) and heart rate recovery kinetics from 1 to 30 min post-exercise (**B**) for guarana and placebo conditions. a: significantly different between conditions (*p* < 0.05).

For HR, a significant main effect of Group (F = 209.64, *p* < 0.001, η^2^p = 0.43) and Time (F = 20,083.4, *p* < 0.001, η^2^p = 0.99), as well as a significant Group × Time interaction (F = 6.54, *p* < 0.001, η^2^p = 0.09), were observed. The Guarana condition was associated with significantly higher mean HR values throughout the SSG bouts compared to placebo (Figure 4A). Regarding HR recovery, a significant main effect of Time (F = 4613.24, *p* < 0.001, η^2^p = 0.99) and a significant Group × Time interaction (F = 14.57, *p* < 0.001, η^2^p = 0.17) were detected. Guarana resulted in significantly higher absolute HR values (slower initial recovery) at 1 and 2 min post-exercise (*p* < 0.01). However, guarana exhibited significantly lower HR values at 15 and 30 min post-exercise compared to PLA (*p* < 0.01; Figure 4B). No significant differences were observed for the RPE recorded 30 min post-exercise (guarana: 6.31 ± 0.57 vs. placebo: 6.29 ± 0.55; *p* = 0.94).

### 3.4. Subjective and Psychological Outcomes

**Figure 5:**
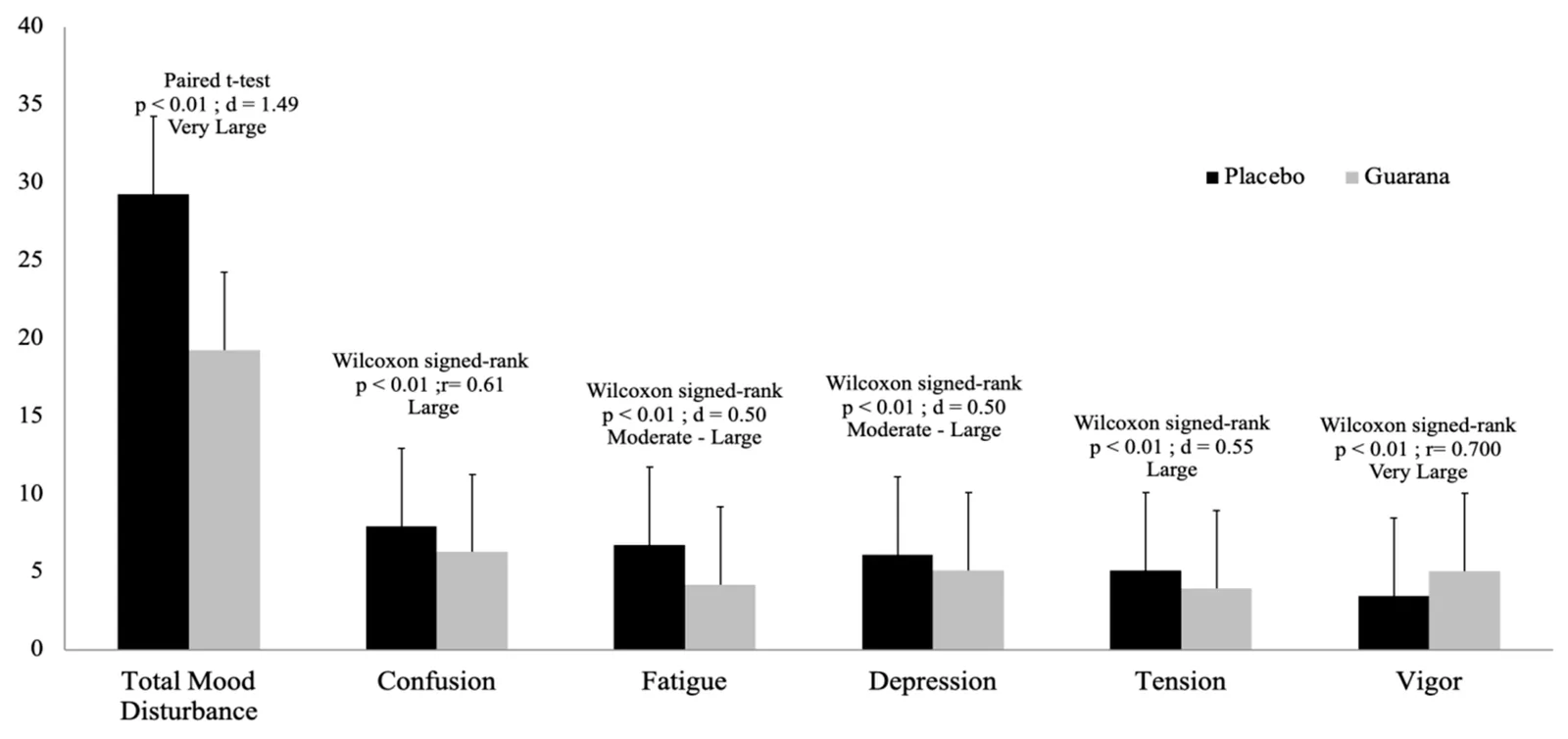
Effect of guarana compared to placebo on Profile of Mood States.

Mental effort was significantly lower in the guarana condition compared with placebo at both pre-exercise (3.00 ± 0.82 vs. 4.00 ± 0.73; *p* < 0.01, r = 0.53) and post-exercise (5.00 ± 0.82 vs. 6.19 ± 0.75; *p* < 0.01, r = 0.59). Guarana significantly enhanced the psychological profile prior to the SSG. TMD was significantly lower at pre-exercise following guarana ingestion compared with placebo. Notable improvements were seen in Vigor–Activity and reductions in Confusion and Tension (Figure 5). The Hooper Index 24 h post-exercise showed no significant differences for fatigue (d = 0.09), sleep quality (d = 0.00), or muscle soreness (d = −0.01). The total score was nearly identical between conditions (12.25 ± 3.52 vs. 12.53 ± 3.24; d = −0.06).

## 4. Discussion

The present study examined the acute effects of guarana supplementation (300 mg) on attentional performance, executive function, technical skills, physiological responses, and psychological state in soccer players during high-intensity SSGs. The main finding was that guarana significantly enhanced basic attentional processing and reduced perceived mental exertion both before and after the exercise protocol. Furthermore, mood states were significantly improved prior to exercise, with large effect sizes observed for vigor and reduced confusion. Results showed that HR remained higher throughout exercise and recovery in the guarana condition, yet RPE remained identical to placebo. Nevertheless, the LSPT showed a significant improvement pre-exercise followed by a maintenance of technical proficiency post-SSG compared to baseline, demonstrating that while the guarana condition improved technical execution under resting conditions, this specific ergogenic advantage was minimized following high-intensity physical exertion. No significant effects were observed for executive function or recovery on the day after. Taken together, these findings suggest that acute guarana supplementation was associated with reduced perceived mental exertion and improved pre-exercise technical performance, although these effects were not sustained following high-intensity exercise.

Previous investigations of the cognitive effects of guarana have yielded mixed findings. While some studies report improvements in reaction time and alertness [15,18], others suggest that the benefits are largely attributable to caffeine content alone [16,17]. Such discrepancies are likely due to methodological variations, including dosage, absorption kinetics, habitual caffeine consumption, and the specific cognitive parameters assessed [16,22]. In the present study, guarana significantly enhanced TMT-A performance, reflecting improved basic attentional processing and visuomotor speed. This aligns with earlier work showing cognitive benefits at similar doses [18,30,31]. Conversely, no significant improvement was observed for TMT-B, which measures higher-order cognitive flexibility and set-shifting. This finding suggests that guarana’s acute actions are task-dependent, displaying greater sensitivity for simple attentional tasks than for complex executive functions. Likewise, the relatively low caffeine dose provided (~78 mg) may have been sufficient to enhance basic attentional processes but insufficient to influence higher-order executive control functions. Accordingly, the significant main effect of time observed across both conditions suggests a substantial learning effect on the TMT-B, which may have obscured potential inter-group differences [32]. Consequently, the term “cognitive enhancement” should be interpreted with caution; the observed benefits were localized to basic attentional processing rather than higher-order executive control, a distinction consistent with recent meta-analytical perspectives [21].

The LSPT is recognized for its high reliability and discriminant validity across various skill levels [24]. In the present study, a significant Group × Time interaction was observed for LSPT total performance time. Prior to the physical workload, soccer-specific technical execution was significantly enhanced in the guarana condition compared with placebo (6.2% faster; *p* < 0.01), indicating a marked acute ergogenic effect. However, following exercise, this between-condition difference was completely attenuated (0.5% difference; 53.75 ± 3.67 s vs. 53.46 ± 6.36 s; *p* > 0.05). These findings suggest that low-dose guarana supplementation improves ball control and execution speed under rested conditions, whereas this advantage is largely abolished after high-intensity exertion induced by SSGs. The Yerkes–Dodson law [33] provides a plausible psychophysiological explanation for this pattern. The moderate increase in central nervous system arousal induced by the low caffeine content of guarana likely optimized the arousal–performance relationship before exercise, thereby facilitating cognitive processing and psychomotor coordination. However, the substantial fatigue and physiological stress generated by repeated high-intensity SSG bouts may have pushed arousal beyond the optimal range or impaired central neural drive, thereby eliminating the pre-exercise technical benefit. According to this model, performance is maximized at moderate levels of arousal and declines when arousal becomes either insufficient or excessive. In the present study, simple cognitive processing (TMT-A) appeared to benefit from increased arousal, whereas complex and highly precise technical skills assessed by the LSPT required a more refined balance that was disrupted after exercise. Moreover, guarana contains several bioactive compounds, including theobromine, theophylline, and tannins [16], which may modulate caffeine pharmacokinetics by slowing absorption and potentially promoting a more sustained stimulatory effect throughout the SSG protocol.

The ergogenic effect of low-dose guarana supplementation on HR responses cannot be attributed to variances in physical workload, given that the SSG protocol was rigorously standardized and identical across both experimental trials. Instead, the observed HR profile reflects a distinct biphasic kinetic response. Guarana significantly increased mean HR throughout the SSG bouts and altered recovery kinetics, characterized by an initial central stimulatory elevation and slower recovery during the early acute phase (1–2 min), likely due to sustained sympathetic drive. Nonetheless, this early delay was followed by an accelerated and more efficient cardiorespiratory down-regulation, characterized by a significantly faster late recovery (15–30 min) compared to the placebo condition. This distinct pattern of post-exercise HR recovery might be mediated by an accelerated parasympathetic reactivation combined with a progressive sympathetic withdrawal [16]. These physiological and kinetic findings align closely with recent investigations demonstrating the targeted ergogenic efficacy of low-dose caffeine protocols in competitive soccer cohorts [34]. Additionally, our results support the growing body of literature highlighting the operational effectiveness of alternative or rapid-delivery caffeinated matrices in elite athletic populations [35], reinforcing that strategic, lower-threshold dosing can yield significant performance preservation without systemic over-stimulation.

Interestingly, despite the significantly elevated HR response during the SSG bouts and the modified early recovery kinetics following guarana supplementation, the overall RPE remained unchanged between conditions. This dissociation suggests that RPE, which is primarily influenced by central motor command, was not increased by the greater physiological activation induced by guarana [36]. Although the physiological demand was higher, the subjective perception of physical effort remained unchanged. Furthermore, the reduction in PME without a corresponding change in RPE suggests that the mechanisms underlying mental and physical effort perception are partially distinct [37]. In the guarana condition, the “top-down” optimization of cognitive resources may have reduced the mental strain required to navigate the complex environment of the SSG, effectively decoupling cognitive fatigue from peripheral metabolic signals [37]. TMD was significantly lower following guarana ingestion, with very large effects on Vigor (r = 0.70). This indicates that guarana induces a more positive psychological state prior to exercise, reflecting enhanced mental readiness. Notably, this mood enhancement occurred alongside the onset of cardiovascular activation, suggesting that guarana’s psychological and sympathetic effects emerge simultaneously. Despite these acute changes, no differences were found in the Hooper Index 24 h post-session. Sleep quality, stress, and muscle soreness were comparable between conditions. The absence of delayed impairment is clinically relevant, suggesting that a 300 mg dose of guarana may provide acute benefits without compromising overnight recovery, likely because it was ingested in the morning. This aligns with the known pharmacokinetic profile of caffeine, which is typically cleared within 12–24 h [15]. Importantly, no side effects were reported compared with placebo, further supporting the tolerability of the supplementation protocol.

Several methodological limitations should be acknowledged when interpreting the present findings. First, the sample included only male soccer players, limiting the generalizability of the results to female players and other athletic populations. Second, although the 20 min 4 vs. 4 small-sided game protocol was sport-specific and physiologically demanding, it may not fully replicate the cumulative physical, cognitive, and technical demands experienced during a 90 min competitive match. Third, because habitual caffeine intake was not available as a covariate, the potential moderating role of baseline caffeine-use patterns on individual responses to guarana could not be examined. In addition, CYP1A2 genetic variation (rs762551), which may influence caffeine metabolism and individual responsiveness to caffeine-containing substances, was not assessed [15,18,22]. Lastly, the absence of a third condition involving anhydrous caffeine matched to the caffeine dose provided by guarana prevented the isolation of caffeine-specific effects from the potential contribution of other bioactive compounds present in guarana. Therefore, the observed cognitive and technical effects may reflect caffeine, non-caffeine constituents of guarana, or their combined action. Although the observed effects were modest and should be interpreted cautiously in relation to overall match-play performance, future studies should use multi-arm designs including placebo, caffeine-matched anhydrous caffeine, and guarana conditions. Such studies should also include broader athlete populations, systematically quantify habitual caffeine intake, and consider genetic profiling to better characterize interindividual responsiveness and refine personalized supplementation strategies in soccer players.

From a field and physiological perspective, the practical application of these findings requires careful integration by sports nutritionists and conditioning specialists. Given that the technical optimization induced by acute guarana supplementation (300 mg) was primarily confined to the pre-exercise baseline (6.2% faster LSPT execution) and did not persist following the high-intensity intermittent SSG bouts, its timing should be considered strategically. Coaches should consider the timing of supplementation relative to specific training blocks; rather than expecting a prolonged ergogenic effect across an entire 90 min competitive match, this low-dose protocol may be best utilized to sharpen attentional readiness and technical precision during structured pre-match warm-ups, or immediately prior to shorter, high-density tactical drills where cognitive and skill demands are particularly high. The present findings suggest that acute guarana supplementation (~300 mg, providing approximately 78 mg of caffeine) administered 60–75 min before high-intensity soccer-specific activity may help enhance attentional readiness, improve pre-exercise mood state, reduce perceived mental exertion, and optimize technical performance during the initial phases of activity. Because this technical optimization is concentrated around the pre-exercise window, coaches may consider scheduling high-accuracy technical drills immediately following the absorption period when the initial arousal response is optimal. Given the absence of reported side effects compared with placebo and the absence of detectable adverse changes in next-day wellness indicators (Hooper Index), morning guarana supplementation at the tested dose appears to be well tolerated within this sample of male amateur players.

## 5. Conclusions

In conclusion, acute ingestion of a low-dose guarana extract (~300 mg) before soccer-specific activity was associated with a modest improvement in TMT-A performance, reflecting enhanced basic attentional processing. No significant effect was observed for TMT-B, indicating that higher-order executive function was not influenced. Guarana supplementation was also associated with improved pre-exercise LSPT performance (6.2% faster execution time), but this advantage was not maintained after the high-intensity SSG protocol. Physiologically, guarana increased mean exercise heart rate and slowed early recovery kinetics, while perceived mental exertion was reduced and mood state improved. No meaningful effects were observed on 24 h wellness indices. Overall, these findings suggest that low-dose guarana may represent a potential pre-exercise nutritional strategy to support basic attentional readiness and technical performance under soccer-specific conditions, without increasing perceived physical exertion (RPE) or adversely affecting short-term recovery.

## Figures and Tables

**Table 1:** Summary of mixed-model ANOVA results (F-values, *p*-values, and η^2^p) for Group, Time, and Group × Time interaction effects across cognitive, technical, and physiological outcomes.

Parameters	Condition Effect			Time Effect			Interaction (Group × Time)		
	F	*p*	η^2^p [90% CI]	F	*p*	η^2^p [90% CI]	F	*p*	η^2^p [90% CI]
**TMT-A**	7.97	0.005	0.05 [0.01, 0.12]	4.16	0.02	0.05 [0.01, 0.12]	0.44	0.64	0.01 [−0.02, 0.06]
**TMT-B**	3.07	0.08	0.02 [−0.01, 0.08]	10.37	0.01	0.12 [0.04, 0.22]	0.55	0.57	0.01 [−0.02, 0.06]
**Raw Time**	6.63	0.01	0.04 [0.01, 0.10]	119.05	<0.001	0.61 [0.52, 0.69]	7.00	0.01	0.08 [0.02, 0.17]
**Penalty Time**	0.46	0.50	0.00 [−0.02, 0.04]	20.43	<0.001	0.21 [0.12, 0.31]	0.37	0.69	0.01 [−0.02, 0.06]
**LSPT**	3.03	0.08	0.02 [−0.01, 0.08]	103.41	<0.001	0.57 [0.48, 0.65]	4.96	0.01	0.06 [0.01, 0.15]
**HR**	209.64	<0.001	0.43 [0.37, 0.49]	20,083.4	<0.001	1.00 [0.999, 1.000]	6.54	<0.001	0.09 [0.04, 0.17]
**HR rec**	1.94	0.17	0.01 [−0.01, 0.05]	4613.24	<0.001	0.99 [0.98, 0.99]	14.57	<0.001	0.17 [0.09, 0.26]

η^2^p = partial eta squared. 90% confidence intervals for η^2^p were calculated using the noncentral F distribution method (Smithson, 2001). CI values that include zero indicate non-significant effects (*p* ≥ 0.05). TMT-A: Trail Making Test Part A; TMT-B: Trail Making Test Part B; LSPT: Loughborough Soccer Passing Test; HR: heart rate; HR rec: heart rate recovery.

## Data Availability

Data are contained within the article.
